# Tailored acoustic metamaterials. Part II. Extremely thick-walled Helmholtz resonator arrays

**DOI:** 10.1098/rspa.2022.0125

**Published:** 2022-06

**Authors:** Michael J. A. Smith, I. David Abrahams

**Affiliations:** Department of Applied Mathematics and Theoretical Physics, University of Cambridge, Wilberforce Road, Cambridge CB3 0WA, UK

**Keywords:** acoustics, Helmholtz resonator, two-dimensional array, matched asymptotic expansions, multipole methods, metamaterial

## Abstract

We present a solution method which combines the technique of matched asymptotic expansions with the method of multipole expansions to determine the band structure of cylindrical Helmholtz resonator arrays in two dimensions. The resonator geometry is considered in the limit as the wall thickness becomes very large compared with the aperture width (the *extremely thick-walled* limit). In this regime, the existing treatment in Part I (Smith & Abrahams, 2022 Tailored acoustic metamaterials. Part I. Thin- and thick-walled Helmholtz resonator arrays), with updated parameters, is found to return spurious spectral behaviour. We derive a regularized system which overcomes this issue and also derive compact asymptotic descriptions for the low-frequency dispersion equation in this setting. We find that the matched-asymptotic system is able to recover the first few bands over the entire Brillouin zone with ease, when suitably truncated. A homogenization treatment is outlined for describing the effective bulk modulus and effective density tensor of the resonator array for all wall thicknesses. We demonstrate that *extremely thick-walled* resonators are able to achieve exceptionally low Helmholtz resonant frequencies, and present closed-form expressions for determining these explicitly. We anticipate that the analytical expressions and the formulation outlined here may prove useful in designing metamaterials for industrial and other applications.

## 1. Introduction

**Figure 1.**
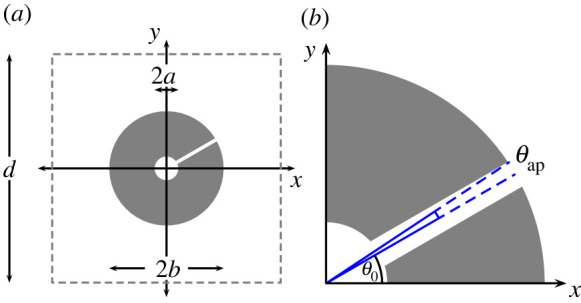
(*a*) Representative fundamental unit cell in non-dimensional coordinates for a square array of Helmholtz resonators with high aspect ratios (i.e. *extremely thick-walled* Helmholtz resonators), where a and b denote the inner and outer radii, respectively, and d is the periodicity; (*b*) close-up of neck region showing central aperture angle $\theta_{0}$ and aperture half-width angle $\theta_{\text{ap}}$. (Online version in colour.)

In Part I of this study [1], we outlined a matched asymptotic-multipole treatment for determining the band structure of thin- and moderately thick-walled Helmholtz resonator arrays. However, this formulation implicitly assumed that the wall thickness was not too large as compared with the aperture width; an assumption that prevents us from achieving very low Helmholtz resonance frequencies, or equivalently, very low first-band gaps. Here we consider an important extension to the results derived in Part I by examining arrays of *extremely thick-walled* resonators (i.e. those with very high aspect ratios of neck length to neck width) to achieve a low frequency resonance, see figure 1. We also discuss a homogenization procedure for all wall thickness configurations. For reference, the nomenclature *split-ring resonator* is often used for the design considered here, as well as *loop-gap resonator* or *split-tube resonator* from across the literature.

On the topic of homogenization, the literature on two- and three-dimensional Helmholtz resonator arrays is extensive, particularly since the resonator can exhibit (1) singular behaviour with respect to its geometry, as well as (2) singular behaviour with respect to a constitutive quantity (e.g. a high-contrast conductivity). We do not attempt to conduct an exhaustive literature review here but will instead highlight key works of interest. To clarify by means of example, a geometrically singular medium could include an array of resonators where the aperture width of each resonator contracts much more rapidly than some other geometric parameter, as wavelengths become long relative to the period of the unit cell. Interestingly, existing works in acoustics [2,3] suggest that for the *extremely thick-walled* Helmholtz resonator array problem, the effective density is not frequency dependent, whereas the effective bulk modulus is indeed frequency dependent and takes negative values for a fixed frequency interval. This very same behaviour is observed in two-scale asymptotic treatments of high contrast arrays of cylinders for the Helmholtz equation [4]. The observation that frequency dependence emerges only in the effective bulk modulus is consistent with the assertion in Haberman & Guild [5] that a dynamic *compressibility* is observed near the Helmholtz resonance frequency for Helmholtz resonator arrays, and not a dynamic *density* response.

The outline of this paper is as follows. First, we briefly restate the governing equations offered in Part I in the following section. We then determine the leading-order outer and inner solutions within the neck region in §2, where asymptotic matching is also conducted. In §3, we construct the regularized system for *extremely thick-walled* resonator arrays before constructing asymptotic dispersion equations in §4. In §5, we present closed-form representations for the Helmholtz resonance/cutoff frequency. Numerical examples are then considered in §7, and finally an extended discussion is given in §8, which highlights the differences of the model results presented here to those presented in Part I.

### (a) Governing wave equation

We consider wave propagation in an acoustic medium satisfying the scalar wave equation

1.1 $$(\partial_{\bar{x}}^{2}+\partial_{\bar{y}}^{2})\;\bar{\phi }+k^{2}\bar{\phi }=0,$$

where $\bar{x}$ denotes dimensional Cartesian coordinates, $k^{2}=\rho \omega^{2}/B$ is the square of the wavenumber, B is the bulk modulus, ρ is the mass density of the medium, $\bar{\phi }$ is the velocity potential, and $\partial_{\bar{x}}$ denotes a partial derivative with respect to $\bar{x}$, for example (see Part I for further details). Within this acoustic medium, we immerse a two-dimensional square array of rigid resonators as shown in figure 1, spaced a distance $\bar{d}$ apart, satisfying Neumann conditions on the wall faces, and Bloch conditions throughout the cell. Implicitly we examine time-harmonic solutions of the form exp⁡(−iωt) where ω is the angular frequency, but this factor is suppressed throughout for ease of exposition.

## 2. Helmholtz resonators in the extremely thick-walled limit

We shall now employ the method of matched asymptotic expansions [6,7] as outlined in Part I, where through non-dimensional rescaling we introduced *inner* and *outer* regions of the unit cell. However, in contrast to the treatment of thick-walled resonators in Part I, where we partitioned the unit cell for the outer problem into two domains, we now partition the unit cell for the outer problem into three domains: the interior, neck and exterior regions, and consider the solution in each domain. The outer solutions for the interior and exterior regions are identical to those presented in Part I (except that the interior region now has a different radius to the exterior region), whose results we restate for reference below. For the outer domains, we use coordinates scaled on the wavenumber

2.1 $$x=k\bar{x}\;\text{and}\;y=k\bar{y},$$

with the inner and outer radii of the resonator, $\bar{a}$ and $\bar{b}$, scaled as $a=k\bar{a}$ and $b=k\bar{b}$, respectively, the lattice period $\bar{d}$ is scaled as $d=k\bar{d}$, the non-dimensional cylinder thickness is 2m=b−a, and the aperture half-width $\bar{\ell }$ is scaled as $\varepsilon =k\bar{\ell }$. In all that follows we take the asymptotic limit ε→0. We now examine the outer solution in the neck region in detail.

### (a) Outer solution in the neck region

Note that from the unit cell configuration shown in figure 1, we first rotate and translate the lattice as $\left(\tilde{x},\tilde{y})\mapsto (x\sin\theta_{0}-y\cos\theta_{0},x\cos\theta_{0}+y\sin\theta_{0}-b\right)$, where $\theta_{0}$ is the central aperture angle, so that the exterior mouth of the resonator is located at $\left(\tilde{x},\tilde{y})=(0,0\right)$. In this coordinate setting, we solve

**Figure 2.**
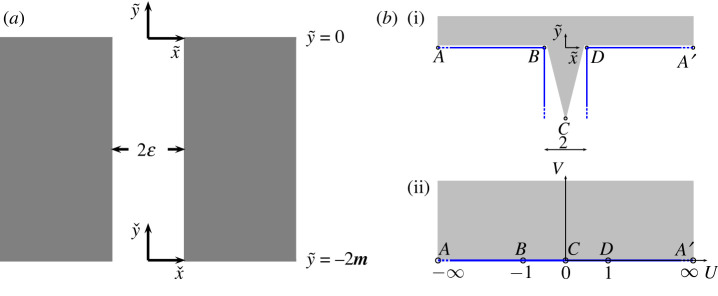
(*a*) Outer problem inside the thick-walled resonator neck of width 2ε and length 2m (not to scale) where $\left(\tilde{x},\tilde{y}\right)$ denotes the centre of the exterior mouth and $\left(\overset{ˇ}{x},\overset{ˇ}{y}\right)$ the centre of the interior mouth. (*b*)(i) Inner problem geometry at the exterior neck entrance and (*b*)(ii) Inner problem geometry after applying the Schwartz–Christoffel mapping (2.7); the capital letters A,…,D and $A^{\prime },\ldots ,D^{\prime }$ denote points of correspondence between the two complex planes. (Online version in colour.)

2.2 $$(\partial_{\tilde{x}}^{2}+\partial_{\tilde{y}}^{2}+1)\phi_{\text{neck}}=0\;\text{for}\;(\tilde{x},\tilde{y})\in S_{N},\;\text{with}\;\partial_{\tilde{x}}\phi_{\text{neck}}{|}_{\tilde{x}=\pm \varepsilon }=0,$$

in the neck region of the resonator $S_{N}=\{(\tilde{x},\tilde{y}):(-\varepsilon ,\varepsilon )\times (-2m,0)\}$ as illustrated in figure 2, in the limit as ε→0. Away from the aperture mouths, (2.2) admits the complete general solution

2.3 $$\begin{array}{rl} & \phi_{\text{neck}}=\sum_{n=0}^{\infty }\{p_{n}{\text{e}}^{\text{i}\lambda_{n}\tilde{y}}+q_{n}{\text{e}}^{-\text{i}\lambda_{n}\tilde{y}}\}\cos\left(\frac{n\pi (\tilde{x}+\varepsilon )}{2\varepsilon }\right),\\ & \;\text{where }\lambda_{n}=\left\{ \begin{array}{ll} \sqrt{1-{\left(\frac{n\pi }{2\varepsilon }\right)}^{2}}, & \frac{n\pi }{2\varepsilon }<1,\\ \text{i}\sqrt{{\left(\frac{n\pi }{2\varepsilon }\right)}^{2}-1}, & \frac{n\pi }{2\varepsilon }>1. \end{array}\right. \end{array}\}$$

In the closing aperture limit, the dominant contribution comes from the n=0 term and so the solution takes the form $\lim_{\varepsilon \to 0}\phi_{\text{neck}}∼p_{0}{\text{e}}^{\text{i}\tilde{y}}+q_{0}{\text{e}}^{-\text{i}\tilde{y}}$. Accordingly, the outer solution asymptotics near the entrance and exit to the neck are given by

2.4a $$\lim_{\tilde{x}\to 0}\lim_{\tilde{y}\to 0}\phi_{\text{neck}}∼(p_{0}+q_{0})+\text{i}\tilde{y}(p_{0}-q_{0}),$$

and

2.4b $$\lim_{\overset{ˇ}{x}\to 0}\lim_{\overset{ˇ}{y}\to 0}\phi_{\text{neck}}∼(p_{0}{\text{e}}^{-2\text{i}m}+q_{0}{\text{e}}^{2\text{i}m})+\text{i}\overset{ˇ}{y}(p_{0}{\text{e}}^{-2\text{i}m}-q_{0}{\text{e}}^{2\text{i}m}),$$

respectively, where we express (2.4*b*) in terms of the shifted origin $\left(\overset{ˇ}{x},\overset{ˇ}{y})=(\tilde{x},\tilde{y}+2m\right)$. We note that for the inner solutions that follow, we use the same rotated and translated frame $\left(\tilde{x},\tilde{y}\right)$, and so we do not need to express the representations (2.4) above in terms of (x,y).

### (b) Outer solution in the interior and exterior regions

We now restate results for the outer solution in the interior and exterior domains (see equation (6.11) of Part I) as we approach the mouths in the form

2.5a $$\lim_{\theta \to \theta_{0}}\lim_{r\to b}\phi_{\text{ext}}∼\frac{2\text{i}A}{\pi }\left[\gamma_{\text{e}}-\frac{\text{i}\pi }{2}+\log\left(\frac{\tilde{r}}{2}\right)\right]+\sum_{n=-\infty }^{\infty }b_{n}Y_{n}(b){\text{e}}^{\text{i}n\theta_{0}}-\sum_{n=-\infty }^{\infty }\left\{\frac{AQ_{n}}{2}+b_{n}Y_{n}^{\prime }(b){\text{e}}^{\text{i}n\theta_{0}}\right\}\frac{J_{n}(b)}{J_{n}^{\prime }(b)},$$

and

2.5b $$\lim_{\theta \to \theta_{0}}\lim_{r\to a}\phi_{\text{int}}∼\frac{2\text{i}B}{\pi }\left[\gamma_{\text{e}}-\frac{\text{i}\pi }{2}+\log\left(\frac{\overset{ˇ}{r}}{2}\right)\right]-\frac{B}{2}\sum_{n=-\infty }^{\infty }\frac{{\overset{ˇ}{Q}}_{n}}{J_{n}^{\prime }(a)}J_{n}(a),$$

where *A*, *b*_*n*_, and *B* are as yet unknown constants, $Q_{n}=J_{n}(b)H_{n}^{(1)\prime }(b)+J_{n}^{\prime }(b)H_{n}^{\left(1\right)}(b)$ and ${\overset{ˇ}{Q}}_{n}=J_{n}(a)H_{n}^{(1)\prime }(a)+J_{n}^{\prime }(a)H_{n}^{\left(1\right)}(a)$, along with $\tilde{r}=\sqrt{{\left(x-b\cos\theta_{0}\right)}^{2}+{\left(y-b\sin\theta_{0}\right)}^{2}}$, $\overset{ˇ}{r}=\sqrt{{\left(x-a\cos\theta_{0}\right)}^{2}+{\left(y-a\sin\theta_{0}\right)}^{2}}$, and a=b−2m. In the above, $J_{n}(z)$ and $Y_{n}(z)$ are Bessel functions of the first and second kind, respectively, $H_{n}^{\left(1\right)}(z)$ are Hankel functions of the first kind, and a prime denotes a derivative with respect to argument, i.e. $J_{n}^{\prime }(b)=\partial_{z}J_{n}(z){|}_{z=b}$. Having obtained asymptotic forms for the outer solution in the interior, neck and exterior regions, we now consider the task of determining inner solutions at the junctions to the resonator neck.

### (c) Inner solutions and asymptotic matching procedure

As before, we first rotate and translate the lattice so that the exterior mouth of the resonator is located at the origin in $\left(\tilde{x},\tilde{y}\right)$ coordinates. We now introduce the inner scaling $\tilde{X}=\tilde{x}/\varepsilon$ and $\tilde{Y}=\tilde{y}/\varepsilon$ along with a regular expansion for ϕ as in Part I; substituting these into the system we obtain the leading-order inner boundary value problem

2.6 $$(\partial_{\tilde{X}}^{2}+\partial_{\tilde{Y}}^{2})\Phi =0\;\text{for }(\tilde{X},\tilde{Y})\in {\tilde{S}}_{M},\;\text{with}\;\partial_{\tilde{N}}\Phi =0\;\text{for }(\tilde{X},\tilde{Y})\in \partial {\tilde{S}}_{M},$$

where $\partial_{\tilde{N}}$ denotes the normal derivative, ${\tilde{S}}_{M}=\{(\tilde{X},\tilde{Y}):|\tilde{X}|\leq 1,-\infty <\tilde{Y}<0\}\cup \{(\tilde{X},\tilde{Y}):\tilde{Y}>0\}$ is the exterior mouth domain shown in figure 2, and for clarity we omit the subscript for $\Phi_{0}$, which denotes the leading term in the inner expansion as ε→0. To obtain a solution, we map the exterior mouth region ${\tilde{S}}_{M}$ to the upper-half plane via the Schwartz–Christoffel mapping

2.7 $$\tilde{Z}(W)=\frac{2}{\pi }[{\left(W^{2}-1\right)}^{1/2}-\text{i}\log({\left(W^{2}-1\right)}^{1/2}+\text{i})+\text{i}\log W],$$

where $\tilde{Z}=\tilde{X}+\text{i}\tilde{Y}$ and W=U+iV, which exhibits the asymptotic behaviours

2.8a $$\lim_{W\to 0}\tilde{Z}(W)∼1+\frac{2\text{i}}{\pi }(1-\log2)+\frac{2\text{i}}{\pi }\log W\;\text{and}\;\lim_{W\to \infty }\tilde{Z}(W)∼\frac{2W}{\pi }.$$

The appropriate solution to Laplace’s equation in the upper-half plane satisfying Neumann conditions along V=0 is given by $\Phi =C_{1}\text{Re}(\log W)+C_{2}$, where $C_{1}$ and $C_{2}$ are as yet unknown and from (2.8) it follows that

2.9a $$\lim_{\begin{array}{c} \tilde{Z}\to \infty \\ \left(UHP\right) \end{array}}\Phi =C_{1}\log\left(\frac{\pi \tilde{R}}{2}\right)+C_{2}\;\text{and}\;\lim_{\begin{array}{c} \tilde{Y}\to -\infty \\ |\tilde{X}|<1 \end{array}}\Phi =C_{1}\left[\frac{\pi \tilde{Y}}{2}-1+\log2\right]+C_{2}.$$

For the interior mouth region, the inner solution Ψ, say, is obtained by a treatment analogous to that outlined above, but now expressed in terms of the inner transformation $\overset{ˇ}{X}=\overset{ˇ}{x}/\varepsilon$ and $\overset{ˇ}{Y}=\overset{ˇ}{y}/\varepsilon =(\tilde{y}+2m)/\varepsilon$, where for clarity we also omit the subscript for $\Psi_{0}$. Accordingly, in this region, from (2.7), we expect the asymptotic behaviour

2.9b $$\lim_{\begin{array}{c} \overset{ˇ}{Z}\to \infty \\ \left(LHP\right) \end{array}}\Psi =C_{3}\log\left(\frac{\pi \overset{ˇ}{R}}{2}\right)+C_{4}\;\text{and}\;\lim_{\begin{array}{c} \overset{ˇ}{Y}\to \infty \\ |\overset{ˇ}{X}|<1 \end{array}}\Psi =C_{3}\left[-\frac{\pi \overset{ˇ}{Y}}{2}-1+\log2\right]+C_{4},$$

where $\overset{ˇ}{Z}=\overset{ˇ}{X}+\text{i}\overset{ˇ}{Y}=\overset{ˇ}{R}\;\exp(\text{i}\overset{ˇ}{\Theta })$ and both $C_{3}$ and $C_{4}$ are as yet unknown constants. Subsequently, the matching procedure [6], at leading order, gives rise to the relations

2.10a $$\lim_{\begin{array}{c} \tilde{R}\to \infty \\ \left(UHP\right) \end{array}}\Phi {|}_{\tilde{R}=\tilde{r}/\varepsilon }=\lim_{\tilde{r}\to 0}\lim_{\theta \to \theta_{0}}\phi_{\text{ext}}\;\text{and}\;\lim_{\tilde{Y}\to -\infty }\Phi {|}_{\tilde{X}=\tilde{x}/\varepsilon ,\tilde{Y}=\tilde{y}/\varepsilon }=\lim_{\tilde{x}\to 0}\lim_{\tilde{y}\to 0}\phi_{\text{neck}}$$

and

2.10b $$\lim_{\begin{array}{c} \overset{ˇ}{R}\to \infty \\ \left(LHP\right) \end{array}}\Psi {|}_{\overset{ˇ}{R}=\overset{ˇ}{r}/\varepsilon }=\lim_{\overset{ˇ}{r}\to 0}\lim_{\theta \to \theta_{0}}\phi_{\text{int}}\;\text{and}\;\lim_{\overset{ˇ}{Y}\to \infty }\Psi {|}_{\overset{ˇ}{X}=\overset{ˇ}{x}/\varepsilon ,\overset{ˇ}{Y}=\overset{ˇ}{y}/\varepsilon }=\lim_{\overset{ˇ}{x}\to 0}\lim_{\overset{ˇ}{y}\to 0}\phi_{\text{neck}}.$$

Thus, matching polynomial orders between (2.4) and (2.9), in addition to logarithmic and non-logarithmic terms between (2.5) and (2.9), allows us to determine, after significant algebra, all coefficients $C_{j}$, as well as the monopole amplitudes $B=2\text{i}A\;{\left(2\text{i}\tau_{1}/\pi +\tau_{2}\tau_{5}\right)}^{-1}/\pi$ and

2.11 $$A=\frac{2}{\pi b{\bar{\bar{h}}}_{\varepsilon }}\sum_{n=-\infty }^{\infty }g_{n},$$

where $g_{n}=b_{n}{\text{e}}^{\text{i}n\theta_{0}}/J_{n}^{\prime }(b)$, and

2.12 $${\bar{\bar{h}}}_{\varepsilon }=\frac{2\text{i}}{\pi }\left[\gamma_{\text{e}}-\frac{\text{i}\pi }{2}-\log\left(\frac{\pi }{\varepsilon }\right)-\left(\frac{2\text{i}}{\pi }\tau_{3}+\tau_{4}\tau_{5}\right){\left(\frac{2\text{i}}{\pi }\tau_{1}+\tau_{2}\tau_{5}\right)}^{-1}\right]-\frac{1}{2}\sum_{n=-\infty }^{\infty }\frac{Q_{n}J_{n}(b)}{J_{n}^{\prime }(b)},$$

along with

2.13a $$\tau_{1}=\frac{2\varepsilon }{\pi }(1-\log2)\sin(2m)-\cos(2m),\;\tau_{4}=-\frac{2\varepsilon }{\pi }(1-\log2)\sin(2m)+\cos(2m),$$

2.13b $$\tau_{2}=-\frac{2\varepsilon }{\pi }\sin(2m),\;\tau_{5}=\frac{2\text{i}}{\pi }\left[\gamma_{\text{e}}-\frac{\text{i}\pi }{2}-\log\left(\frac{\pi }{\varepsilon }\right)\right]-\frac{1}{2}\sum_{n=-\infty }^{\infty }\frac{{\overset{ˇ}{Q}}_{n}}{J_{n}^{\prime }(a)}J_{n}(a)$$

2.13c $$\;\mathrm{and}\;\tau_{3}=\left[\frac{2\varepsilon }{\pi }{\left(1-\log2\right)}^{2}-\frac{\pi }{2\varepsilon }\right]\sin(2m)-2(1-\log2)\cos(2m).$$

Hence we obtain the same multipole eigensystem as in equation (4.13) of Part I, which we express as

2.14 $$\begin{array}{rl} & \frac{\text{i}A}{2}\left(\frac{J_{n}^{\prime }(b)Y_{n}(b)+Y_{n}^{\prime }(b)J_{n}(b)}{J_{n}^{\prime }(b)Y_{n}^{\prime }(b)}+2\sum_{m=-\infty }^{\infty }{\left(-1\right)}^{n+m}S_{m-n}^{Y}({\text{k}}_{B})\frac{J_{m}(b)}{Y_{n}^{\prime }(b)}{\text{e}}^{-\text{i}(m-n)\theta_{0}}\right)\\ & \;\;+g_{n}+\sum_{m=-\infty }^{\infty }{\left(-1\right)}^{m+n}S_{m-n}^{Y}({\text{k}}_{B})\frac{J_{m}^{\prime }(b)}{Y_{n}^{\prime }(b)}{\text{e}}^{-\text{i}(m-n)\theta_{0}}g_{m}=0, \end{array}$$

but with the replacement $h_{\varepsilon }\mapsto {\bar{\bar{h}}}_{\varepsilon }$ as written in (2.12). Thus, it would appear that we have a suitable system of equations for examining arrays of *extremely thick-walled* resonators; however, it turns out that the above formulation exhibits pathological behaviour at frequencies near ${\bar{\bar{h}}}_{\varepsilon }=0$ which occurs at much lower frequencies than for the wall thickness values discussed in Part I. This behaviour is an artefact of the formulation, and not related to any physical resonance, and so we now present a regularization procedure for resolving this issue.

## 3. Regularized multipole system formulation

As indicated in the previous section, although the system in Part I with the replacement $h_{\varepsilon }\mapsto {\bar{\bar{h}}}_{\varepsilon }$ is indeed formally correct, it can return spurious spectral behaviours upon truncating for numerical evaluation (i.e. we may observe incorrect folded bands, and flat band surfaces, that are not part of the genuine spectrum in a neighbourhood around frequencies corresponding to ${\bar{\bar{h}}}_{\varepsilon }=0$). We easily resolve this issue by summing (2.14) over all n to obtain a more numerically stable yet formally equivalent representation for A (cf., to the form (2.11) above) as

3.1 $$A=2\text{i}{\left(E+2\left[\sum_{v=-\infty }^{\infty }J_{v}(b)F_{v}\right]-\text{i}\pi b{\bar{\bar{h}}}_{\varepsilon }\right)}^{-1}\sum_{m=-\infty }^{\infty }J_{m}^{\prime }(b)F_{m}g_{m},$$

where

3.2 $$E=\sum_{u=-\infty }^{\infty }E_{u}\;\text{and}\;F_{m}=\sum_{p=-\infty }^{\infty }\frac{{\left(-1\right)}^{p-m}S_{m-p}^{Y}({\text{k}}_{B}){\text{e}}^{\text{i}(p-m)\theta_{0}}}{Y_{p}^{\prime }(b)},$$

with $E_{u}=[J_{u}^{\prime }(b)Y_{u}(b)+Y_{u}^{\prime }(b)J_{u}(b)]/[J_{u}^{\prime }(b)Y_{u}^{\prime }(b)]$. Substituting the representation for A in (3.1) into (2.14) admits the regularized system

3.3 $$g_{n}+\sum_{m=-\infty }^{\infty }{\left(-1\right)}^{m+n}S_{m-n}^{Y}({\text{k}}_{B})\frac{J_{m}^{\prime }(b)}{Y_{n}^{\prime }(b)}{\text{e}}^{-\text{i}(m-n)\theta_{0}}g_{m}-\frac{\chi_{n}}{H_{\varepsilon }}\sum_{m=-\infty }^{\infty }J_{m}^{\prime }(b)F_{m}g_{m}=0,$$

for all n, where

3.4a $$\chi_{n}=E_{n}+2\sum_{p=-\infty }^{\infty }{\left(-1\right)}^{n+p}S_{p-n}^{Y}({\text{k}}_{B})\frac{J_{p}(b)}{Y_{n}^{\prime }(b)}{\text{ e}}^{-\text{i}(p-n)\theta_{0}},$$

and

3.4b $$H_{\varepsilon }=E+2\left[\sum_{v=-\infty }^{\infty }J_{v}(b)F_{v}\right]-\text{i}\pi b{\bar{\bar{h}}}_{\varepsilon }.$$

**Figure 3.**
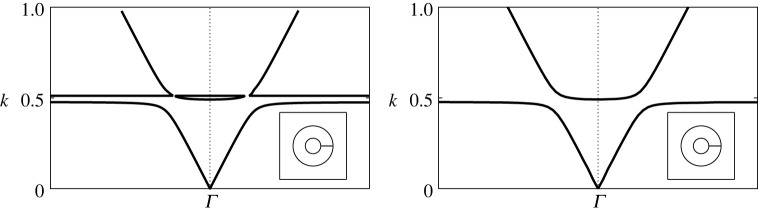
Comparison of band diagrams (near the Γ point) for a two-dimensional square array of extremely thick-walled Helmholtz resonators obtained using multipole methods. Figure (*a*) shows a spurious result obtained using the system (2.14) with the updated ${\bar{\bar{h}}}_{\varepsilon }$, (2.12), and (*b*) shows the correct result obtained using the regularized system (3.3). Inset: corresponding fundamental unit cells. Results are given for a dipole truncation L=1 and truncations $L_{\chi_{n}}=L_{F_{m}}=L_{H_{\varepsilon }}=13$ with $\bar{d}=1$, $\theta_{0}=0$, $\bar{b}=0.3$, h=100 and $\theta_{\text{ap}}=\pi /1024$.

On examining the system (3.3) numerically, we find that spurious effects are removed and the genuine spectrum is observed, see for example figure 3 which is discussed in further detail below. In order for the regularization to be effective, the known expressions E, $\chi_{n}$, $F_{m}$ and $H_{\varepsilon }$ must be suitably converged with appropriately chosen truncation numbers for the sums, which we represent by $L_{E}$, $L_{\chi_{n}}$, $L_{F_{m}}$ and $L_{H_{\varepsilon }}$, respectively.

## 4. Asymptotic representations for the dispersion equation

In this section, we construct an asymptotic representation of the dispersion equation for the regularized system (3.3) at low frequencies. We begin by observing that the lattice sums $S_{m}^{Y}$, which feature in (3.3) as well as in the functions $F_{m}$ (3.2), $\chi_{n}$ (3.4*a*) and $H_{\varepsilon }$ (3.4*b*), can be expressed as

4.1 $$S_{m}^{Y}\approx \sum_{r=-\Omega_{m}}^{\infty }\beta_{r}^{\left(m\right)}b^{r},$$

where $\Omega_{m}=2[\text{Floor}\{(m-3)/4\}+\text{Floor}\{(m-4)/4\}+2]$ for m≥4, denotes the first non-zero order of the lattice sum (i.e. $\Omega_{4}=4$ and $\Omega_{7}=6$), along with $\Omega_{0}=\Omega_{1}=\Omega_{2}=\Omega_{3}=2$ [8]. Closed-form expressions for the first few lattice sums, i.e. the values $\beta_{r}^{\left(m\right)}$ for small m, are derived and presented in appendix A for reference. Note that these terms may contain logarithmic behaviour in the lattice spacing d and therefore in b should the area fraction $f=\pi b^{2}/d^{2}$ be fixed. With the lattice sum expansions (4.1), we examine the system (3.3) in the limit of vanishing b and to within a dipole truncation in n, which admits the matrix form

4.2 $$(H_{\varepsilon }\text{C}-\text{P}-\text{Q})g\approx 0,$$

where C is the matrix representation of the system for an array of Neumann cylinders (obtained by setting $\chi_{n}=0$ in (3.3), see [9, eqn (3.120)]), P and Q are perturbation matrices due to the aperture, and g is the vector of $g_{m}$ coefficients. These matrices take the forms

4.3a $$\begin{array}{rl} & \text{C}\approx \left[\begin{array}{ccc} 1+\frac{\pi }{4}\beta_{-2}^{\left(0\right)} & \frac{\pi b}{4}{\text{e}}^{\text{i}\theta_{0}}{\left(\beta_{-2}^{\left(1\right)}\right)}^{*} & -\frac{\pi }{4}{\text{e}}^{2\text{i}\theta_{0}}{\left(\beta_{-2}^{\left(2\right)}\right)}^{*}\\ -\frac{\pi }{4b}{\text{e}}^{-\text{i}\theta_{0}}\beta_{-2}^{\left(1\right)} & 1-\frac{\pi }{4}\beta_{-2}^{\left(0\right)} & \frac{\pi }{4b}{\text{e}}^{\text{i}\theta_{0}}{\left(\beta_{-2}^{\left(1\right)}\right)}^{*}\\ -\frac{\pi }{4}{\text{e}}^{-2\text{i}\theta_{0}}\beta_{-2}^{\left(2\right)} & -\frac{\pi b}{4}{\text{e}}^{-\text{i}\theta_{0}}\beta_{-2}^{\left(1\right)} & 1+\frac{\pi }{4}\beta_{-2}^{\left(0\right)} \end{array}\right], \end{array}$$

4.3b $$\begin{array}{rl} & \text{P}\approx \left[\begin{array}{ccc} \frac{\pi^{2}|\beta_{-2}^{\left(1\right)}{|}^{2}}{4b} & \frac{\pi^{2}{\text{e}}^{\text{i}\theta_{0}}{\left(\beta_{-2}^{\left(1\right)}\right)}^{*}\beta_{-2}^{\left(0\right)}}{4} & -\frac{\pi^{2}{\text{e}}^{2\text{i}\theta_{0}}{\left({\left(\beta_{-2}^{\left(1\right)}\right)}^{*}\right)}^{2}}{4b}\\ -\frac{\pi (\pi \beta_{-2}^{\left(0\right)}-2){\text{e}}^{-\text{i}\theta_{0}}\beta_{-2}^{\left(1\right)}}{4b^{2}} & -\frac{\pi \beta_{-2}^{\left(0\right)}(\pi \beta_{-2}^{\left(0\right)}-2)}{4b} & \frac{\pi (\pi \beta_{-2}^{\left(0\right)}-2){\text{e}}^{\text{i}\theta_{0}}{\left(\beta_{-2}^{\left(1\right)}\right)}^{*}}{4b^{2}}\\ -\frac{\pi^{2}{\text{e}}^{-2\text{i}\theta_{0}}{\left(\beta_{-2}^{\left(1\right)}\right)}^{2}}{4b} & -\frac{\pi^{2}{\text{e}}^{-\text{i}\theta_{0}}\beta_{-2}^{\left(0\right)}\beta_{-2}^{\left(1\right)}}{4} & \frac{\pi^{2}|\beta_{-2}^{\left(1\right)}{|}^{2}}{4b} \end{array}\right], \end{array}$$

4.3c $$\begin{array}{rl} \mathrm{and}\; & \text{Q}\approx \left[\begin{array}{ccc} \frac{\pi^{2}{\text{e}}^{-\text{i}\theta_{0}}\beta_{-2}^{\left(1\right)}}{2}\eta^{*}+{\text{e}}^{\text{i}\theta_{0}}\pi^{2}{\left(\beta_{-2}^{\left(1\right)}\right)}^{*}\eta & \frac{\pi^{2}b}{2}\beta_{-2}^{\left(0\right)}\eta^{*} & \frac{{\text{e}}^{\text{i}\theta_{0}}\pi^{2}{\left(\beta_{-2}^{\left(1\right)}\right)}^{*}}{2}\eta^{*}\\ \frac{\pi \eta }{b}(2-\pi \beta_{-2}^{\left(0\right)}) & 0 & \frac{\pi \eta^{*}}{b}(2-\pi \beta_{-2}^{\left(0\right)})\\ -\frac{\pi^{2}{\text{e}}^{-\text{i}\theta_{0}}\beta_{-2}^{\left(1\right)}}{2}\eta & \frac{\pi^{2}b}{2}\beta_{-2}^{\left(0\right)}\eta & -\frac{\pi^{2}{\text{e}}^{\text{i}\theta_{0}}{\left(\beta_{-2}^{\left(1\right)}\right)}^{*}}{2}\eta -{\text{e}}^{-\text{i}\theta_{0}}\pi^{2}\beta_{-2}^{\left(1\right)}\eta^{*} \end{array}\right], \end{array}$$

where second-order terms are taken in most matrix entries above for our analysis in the following section, but are omitted here for compactness, and

4.4 $$\eta =-\frac{\beta_{-2}^{\left(0\right)}}{4}+\frac{1}{4}{\text{e}}^{-2\text{i}\theta_{0}}\beta_{-2}^{\left(2\right)}+\sum_{q=1}^{\infty }\left\{\frac{\beta_{-2q-2}^{\left(2q+2\right)}{\text{e}}^{-(2q+2)\text{i}\theta_{0}}}{4^{q+1}(2q+1)!}\right\},$$

with ∗ denoting the complex conjugate operation. In addition, we have

4.5 $$H_{\varepsilon }\approx \frac{-2+4{\bar{\bar{f}}}_{\varepsilon }+\pi \beta_{-2}^{\left(0\right)}}{b}-\frac{\pi }{2}\left[{\text{e}}^{\text{i}\theta_{0}}{\left(\beta_{-2}^{\left(1\right)}\right)}^{*}-\beta_{-2}^{\left(1\right)}{\text{e}}^{-\text{i}\theta_{0}}\right]+O(b),$$

where we take an analogous scaling to that found in Part I in the form ${\bar{\bar{f}}}_{\varepsilon }=\pi b^{2}{\bar{\bar{h}}}_{\varepsilon }/(4\text{i}),$ as well as a dipole truncation for ${\bar{\bar{h}}}_{\varepsilon }$ in (2.12) to obtain the asymptotic representation

4.6a $$\begin{array}{rl} \lim_{b\to 0}\lim_{a\to 0}\lim_{m\to 0}{\bar{\bar{h}}}_{\varepsilon }∼\frac{2\text{i}}{\pi } & \{\frac{1}{b^{2}}-\frac{1}{8}-\log\left(\frac{\pi b}{2\varepsilon }\right)+\left[\frac{1}{a^{2}}-\frac{m\pi }{\varepsilon }-\frac{17}{8}-\log\left(\frac{\pi a}{8\varepsilon }\right)\right]\\ & \;\;\;\times {\left[1+\frac{4\varepsilon m}{\pi }\left(\frac{1}{a^{2}}-\frac{1}{8}-\log\left(\frac{\pi a}{2\varepsilon }\right)\right)\right]}^{-1}\}. \end{array}$$

With the above expansions, we are now able to construct closed-form representations for the dispersion equation of *extremely thick-walled* resonators, however before proceeding to this task, we comment that certain $\beta_{-2n}^{\left(2n\right)}$ terms in (4.4) are vanishing for square lattices, such as $\beta_{-6}^{\left(6\right)}$, $\beta_{-10}^{\left(10\right)}$ and $\beta_{-14}^{\left(14\right)}$ and that many terms are readily extracted from the explicit forms in appendix A.

### (a) Dispersion equation forms along selected symmetry directions

It is instructive to consider cases where the resonator geometry has natural symmetry. Thus, with the asymptotic system (4.2) in mind, we now take $\theta_{0}=0$ and consider Bloch coordinates located on the high symmetry planes of the Brillouin zone, where $\left(k_{B},\theta_{B}\right)$ denotes the polar form of the dimensionless Bloch vector $k_{B}$.

For reference, an outline of the symmetry planes for the fundamental cell in reciprocal space (Brillouin zone) is presented in figure 1 of Part I, showing the paths between the high symmetry Bloch vector coordinates Γ=(0,0), X=(π/d,0), M=(π/d,π/d) and Y=(0,π/d). In the first instance, we evaluate the determinant of the system (4.2) along the ΓX (i.e. $\theta_{B}=0$) direction, which to leading order, returns the isotropic result from Part I (equation (4.19)) with the replacement $f_{\varepsilon }\mapsto {\bar{\bar{f}}}_{\varepsilon }$ in the form

4.7 $$k_{B}^{2}=\frac{1+f}{1-f}\left(1-\frac{2f(1-{\bar{\bar{f}}}_{\varepsilon })}{1-2{\bar{\bar{f}}}_{\varepsilon }}\right),$$

where $f=\pi b^{2}/d^{2}$ denotes the area ratio and ${\bar{\bar{f}}}_{\varepsilon }$ is defined via (4.6*a*). By contrast, along the ΓY direction ($\theta_{B}=\pi /2$) we observe a much more complicated form, which emerges from the symmetries of the system matrices

$$\text{C}=\left[\begin{array}{rrr} ∙ & ⋈ & ⊠\\ ⋄ & ⊚ & -⋄\\ ⊠ & -⋈ & ∙ \end{array}\right],\;\text{P}=\left[\begin{array}{rrr} ∙ & ⋈ & -∙\\ ⋄ & ⊚ & -⋄\\ -∙ & -⋈ & ∙ \end{array}\right]\;\mathrm{and}\;\text{Q}=\left[\begin{array}{ccc} ∙ & ⋈ & -⊠\\ ⋄ & 0 & ⋄\\ ⊠ & ⋈ & -∙ \end{array}\right],$$

where symbols are used to represent the symmetry of each matrix *independently* (and does not imply equivalence of values between matrices).

Subsequently, after a significant amount of algebra, we obtain a representation for the dispersion equation along ΓY that appears to be intractable and hence does not provide any useful insight, despite its high symmetry setting ($\theta_{0}=0$ and $\theta_{B}=\pi /2$). For the purposes of compactness, we do not include the explicit form here, however we conclude that incorporating anisotropy to the dispersion equation for Helmholtz resonator arrays *in the extremely thick-walled limit* is a much more formidable task than for the (moderately) thin-walled case, as shown in Part I. The unexpected complexity of the dispersion equation along ΓY can be understood by considering the geometry of the resonator: in the *extremely thick-walled* limit, the neck of each resonator is so thin and long that the resonator is almost invariant under all rotation and reflection operations for the square lattice. Subsequently, the metamaterial may be considered *almost-isotropic*, even as we approach the resonance frequency, and so next-order asymptotic corrections take on much more complicated forms. Numerical investigations confirm much smaller anisotropy in this scaled setting, to the point where the medium may be essentially regarded as isotropic for practical applications. Accordingly, the low-frequency dispersion equation for *extremely thick-walled* resonators may be taken to be the isotropic form (4.7) (which in fact holds for all values of $\theta_{0}$) over the entire Brillouin cell. The effectiveness of this approximation is examined numerically in §7.

## 5. Helmholtz resonance (cut-off) condition

As determined in Part I and from the denominator of (4.7) above, the Helmholtz resonance condition in the *extremely thick-walled* limit is given by $1-2{\bar{\bar{f}}}_{\varepsilon }\approx 0$ and requires careful examination, as such resonators are capable of achieving very low-frequency resonances. Accordingly, we return to the asymptotic form of ${\bar{\bar{h}}}_{\varepsilon }$ in (4.6*a*) and subsequently write $1-2{\bar{\bar{f}}}_{\varepsilon }\approx 0$ in the form

5.1 $$\frac{1}{8}+\log\left(\frac{\pi b}{2\varepsilon }\right)-\left[\frac{1}{a^{2}}-\frac{m\pi }{\varepsilon }-\frac{17}{8}-\log\left(\frac{\pi a}{8\varepsilon }\right)\right]{\left[1+\frac{4\varepsilon m}{\pi }\left(\frac{1}{a^{2}}\right)\right]}^{-1}\approx 0,$$

where $\tau_{5}$ in (2.13) is taken to O(1) in the numerator and to leading order in the denominator. For the purposes of analysis, we do not advise directly solving (5.1) above to determine the conditions for resonance, as it is unclear which terms play a leading role in the small ε limit; instead, we seek the preferred scalings between ε, a (or b), and m that give the lowest frequency resonance.

It is helpful to introduce the scalings $m=\kappa_{m}\varepsilon^{\mu }$ (i.e. where 0<μ<1, ε→0, and $\kappa_{m}=O(1)$), and $a=\kappa_{a}\varepsilon^{\gamma }$ (i.e. where 0<γ<1, ε→0 and $\kappa_{a}=O(1)$) which admits

5.2 $$\frac{1}{8}+\log\left(\frac{\pi [\kappa_{a}\varepsilon^{\gamma -1}+2\kappa_{m}\varepsilon^{\mu -1}]}{2}\right)\;-\frac{\left[(1/\kappa_{a}^{2})\varepsilon^{-2\gamma }-\kappa_{m}\varepsilon^{\mu -1}\pi -17/8-\log(\pi \kappa_{a}\varepsilon^{\gamma -1}/8)\right]}{\left[1+4\kappa_{m}\varepsilon^{1+\mu -2\gamma }/(\pi \kappa_{a}^{2})\right]}\approx 0.$$

The radii $a=\kappa_{a}\varepsilon^{\gamma }$ and $b=\kappa_{a}\varepsilon^{\gamma }+2\kappa_{m}\varepsilon^{\mu }$ must be of the same order, requiring μ≥γ. We now examine the different dominant balance scalings that are possible for the representation (5.2).

### (i) Dominant balance in all three numerator terms

From the form (5.2), we take the scaling 1+μ−2γ>0, where to ensure that the $O(\varepsilon^{-2\gamma })$, $O(\varepsilon^{\mu -1})$ and O(1) terms balance in the numerator, we require γ=0 and μ=1. Accordingly, the Helmholtz resonance condition then takes the form

5.3a $$\frac{9}{4}+2\log\left(\frac{\pi \kappa_{a}}{4\varepsilon }\right)\approx \left[\frac{1}{\kappa_{a}^{2}}-\pi \kappa_{m}\right],$$

as ε→0. Expressing the above in dimensional terms we find

5.3b $$k_{\text{max}}\approx \frac{1}{\bar{a}}{\left[\frac{9}{4}+2\log\left(\frac{\pi \bar{a}}{4\bar{\ell }}\right)+\frac{\pi \bar{m}}{\bar{\ell }}\right]}^{-1/2},$$

as the Helmholtz resonance condition under this dominant balance scaling.

### (ii) Dominant balance in numerator pairs

Another relationship emerges by considering (5.2) in the 1+μ−2γ>0 regime again, which gives

5.4 $$\frac{9}{4}+\log\left(\frac{\pi^{2}\kappa_{a}\varepsilon^{\gamma -1}[\kappa_{a}\varepsilon^{\gamma -1}+2\kappa_{m}\varepsilon^{\mu -1}]}{16}\right)\approx \left[\frac{1}{\kappa_{a}^{2}}\varepsilon^{-2\gamma }-\pi \kappa_{m}\varepsilon^{\mu -1}\right],$$

where balance on the right-hand side is achieved with the scaling $\gamma =\frac{1}{2}(1-\mu )$, which, as μ≥γ, means that μ≥1/3. Turning to the logarithmic argument we see that for μ>1/3, the resonance condition (5.4) takes the form

5.5a $$\frac{9}{4}+\log\left(\frac{\pi^{2}\kappa_{a}^{2}\varepsilon^{-1-\mu }}{16}\right)\approx \left[\frac{1}{\kappa_{a}^{2}}-\pi \kappa_{m}\right]\varepsilon^{\mu -1},$$

whereas for μ=1/3 we observe

5.5b $$\frac{9}{4}+\log\left(\frac{\pi^{2}\kappa_{a}\varepsilon^{-4/3}[\kappa_{a}+2\kappa_{m}]}{16}\right)\approx \left[\frac{1}{\kappa_{a}^{2}}-\pi \kappa_{m}\right]\varepsilon^{-2/3}.$$

Expressing these resonance conditions in terms of dimensional parameters we again find for μ>1/3 the form in (5.3*b*), and for μ=1/3 the slightly different form

5.6 $$k_{\text{max}}\approx \frac{1}{\bar{a}}{\left[\frac{9}{4}+\log\left(\frac{\pi^{2}\bar{a}\bar{b}}{16{\bar{\ell }}^{2}}\right)+\frac{\pi \bar{m}}{\bar{\ell }}\right]}^{-1/2},$$

with numerical investigations suggesting that only minor differences are found between (5.3*b*) and (5.6), since a and b must be the same order. For reference, under the dominant balance scaling μ+1−2γ>0, we may write

5.7 $$\bar{\bar{f_{\varepsilon }}}\approx \frac{b^{2}}{2}\left[\frac{1}{a^{2}}+\frac{1}{b^{2}}-\frac{9}{4}-\frac{\pi m}{\varepsilon }-\log\left(\frac{\pi^{2}ab}{16\varepsilon^{2}}\right)\right],$$

which is identical to the result obtained by considering ${\overset{ˇ}{f}}_{\varepsilon }$ for moderately thick-walled resonators in equation (6.13) of Part I in the limit as $h=\bar{m}/\bar{\ell }\to \infty$, where q≈4exp⁡(−2−πh) and C≈2/π. Note however that we have previously taken m→0 to obtain (4.6*a*), and so such a limit corresponds to a regime where the aperture width 2ε contracts much more rapidly than the aperture length 2m.

## 6. Homogenization of Helmholtz resonator arrays

### (a) Classical homogenization results for arrays of ideal cylinders

For an isotropic fluid medium of density ρ and bulk modulus B, structured with a two-dimensional array of isotropic fluid *cylinders*, of density $\rho_{c}$ and bulk modulus $B_{c}$, the effective density and bulk modulus are given explicitly in the quasi-static limit by [10,11]

6.1 $$\rho_{\text{eff}}^{-1}=\frac{1}{\rho }\frac{\rho_{c}(1-f)+\rho (1+f)}{\rho_{c}(1+f)+\rho (1-f)}\;\text{and}\;B_{\text{eff}}^{-1}=\frac{1-f}{B}+\frac{f}{B_{c}},$$

where $f=\pi b^{2}/d^{2}$, as defined previously, denotes the filling fraction. Subsequently, results corresponding to a two-dimensional array of cylinders with Neumann boundary conditions on the walls take the form

6.2a $$\rho_{\text{eff}}^{-1}=\frac{1-f}{\rho (1+f)}\;\text{and}\;B_{\text{eff}}^{-1}=\frac{1-f}{B},$$

which are obtained via the limits $B_{c}\to \infty$ and $\rho_{c}\to \infty$ in (6.1) above. Substituting (6.2*a*) into the (dimensional) dispersion equation for plane waves in an unbounded isotropic medium

6.2b $${\bar{k}}_{B}\rho_{\text{eff}}^{-1}{\bar{k}}_{B}-\omega^{2}B_{\text{eff}}^{-1}=0,$$

we obtain the (dimensionless) dispersion equation for an array of Neumann cylinders in the form $k_{B}^{2}=1+f$, as seen from our analysis in Part I and from Movchan *et al.* [9, Eq. (3.158)], and where ${\bar{k}}_{B}=kk_{B}$ is the nondimensional Bloch vector. Similarly, if we return to the two-phase fluid array results (6.1) and take the limits $B_{c}\to B$ and $\rho_{c}\to \infty$ we obtain

6.3a $$\rho_{\text{eff}}^{-1}=\frac{1-f}{\rho (1+f)}\;\text{and}\;B_{\text{eff}}^{-1}=\frac{1}{B},$$

which on substitution in (6.2*b*) returns the dispersion relation

6.3b $$k_{B}^{2}=\frac{1+f}{1-f},$$

and is identical to the lowest-order (isotropic) approximation for an array of thin-walled Helmholtz resonators at quasi-static frequencies (i.e. ω→0), as given in eqn (4.22) of Part I and in Llewellyn Smith & Davis [12]. That is, the lowest-order (isotropic) approximation for the dispersion equation of a Helmholtz resonator array (6.3*b*) *at very low frequencies* is indistinguishable from an array of fluid cylinders that do not possess a contrast in bulk modulus but are much denser than the background fluid. On comparing (6.2*a*) and (6.3*a*) we see that the impact of introducing a small gap into the wall of a perfect Neumann cylinder has the leading-order effect of modifying the effective bulk modulus but not the effective density. As such, we may expect to recover the same expressions for the bulk modulus and density in (6.3*a*) at low frequencies from any isotropic descriptions for resonator arrays.

### (b) Isotropic descriptions for thin-walled Helmholtz resonator arrays

Having discussed the behaviour of the isotropic description at zero frequency, we now restate the result obtained in Part I (equation (4.19))

6.4 $$k_{B}^{2}=\frac{1+f}{1-f}\left(1-\frac{2f(1-f_{\varepsilon })}{1-2f_{\varepsilon }}\right),$$

which is valid for frequencies across the range of the first band surface, where

6.5 $$f_{\varepsilon }∼1-\frac{b^{2}}{8}+b^{2}\log\left(\frac{\theta_{\text{ap}}}{2}\right),$$

and $\theta_{\text{ap}}$ denotes the aperture half-width angle. Here, we emphasize that there are infinitely many ways in which this dispersion equation may be decomposed into the form (6.2*b*). For example, we may extract from (6.4) the response functions

6.6 $$\rho_{\text{eff}}^{-1}=\frac{\left(1-f\right)}{\rho (1+f)}\;\text{and}\;B_{\text{eff}}^{-1}=\frac{1}{B}\frac{\left(1-2f_{\varepsilon }-2f(1-f_{\varepsilon })\right)}{\left(1-2f_{\varepsilon }\right)},$$

as candidates for the behaviour of the resonator array, within an isotropic approximation. Such expressions are consistent with the two-phase fluid results (6.3*a*) as ω→0, vanishing filling fraction results f→0, and the expressions for Neumann cylinders (6.2*a*) as the aperture is closed (i.e. as $f_{\varepsilon }\to \infty$). The absence of dispersion in the density response is also consistent with existing literature on the topic [2,3]. Thus, we consider (6.6) as the effective (homogenized) quantities of the medium for frequencies spanning the first band surface, within an isotropic approximation.

### (c) Anisotropic descriptions for thin-walled Helmholtz resonator arrays

As established in Part I, thin-walled resonator arrays generally exhibit strong anisotropy at low frequencies, and so isotropic descriptions are insufficient to accurately describe the first band surface. Accordingly, we restate the anisotropic result for the first spectral band from Part I (equation (4.25*a*)):

6.7 $$k_{B}^{2}=\frac{\left(f+1)[b^{2}f(2f-1)+f_{\varepsilon }(f+1)(2f_{\varepsilon }f-2f_{\varepsilon }-2f+1)\right]}{b^{2}f\cos(2[\theta_{0}-\theta_{B}])+b^{2}f^{2}+f_{\varepsilon }(2f_{\varepsilon }-1)(f^{2}-1)},$$

and emphasize once more that the assignment of the effective density and bulk modulus is non-unique in the dispersion equation for plane waves in an anisotropic medium [13]

6.8 $${\bar{k}}_{Bi}(\rho_{\text{eff}}^{-1}{)}_{ij}{\bar{k}}_{Bj}-\omega^{2}B_{\text{eff}}^{-1}=0.$$

For example, we may propose the candidate forms

6.9a $$\rho_{\text{eff}}^{-1}=\frac{\left(1-f\right)}{\rho (1+f)}\left\{\left[\begin{array}{cc} 1 & 0\\ 0 & 1 \end{array}\right]+\frac{b^{2}f}{H}\left[\begin{array}{cc} -[1-\cos(2\theta_{0})] & \sin(2\theta_{0})\\ \sin(2\theta_{0}) & -[1+\cos(2\theta_{0})] \end{array}\right]\right\}$$

and

6.9b $$B_{\text{eff}}^{-1}=\frac{\left(1-f\right)}{BH}[b^{2}f(2f-1)+(f+1)f_{\varepsilon }(2(f-1)f_{\varepsilon }-2f+1)],$$

where

6.10 $$H=b^{2}f^{2}+b^{2}f+(f^{2}-1)f_{\varepsilon }(2f_{\varepsilon }-1).$$

These expressions are consistent with both the isotropic results (6.6) and the two-phase fluid results (6.3*a*) as ω→0, as well as with the closed aperture (Neumann cylinder) results (6.2*a*) as $f_{\varepsilon }\to \infty$ and the vanishing fill fraction limit f→0. Note that the expression H emerges from the anisotropic approximation to the Helmholtz resonance condition $\det\{\rho_{\text{eff}}^{-1}\}=H=0$.

### (d) Descriptions for moderately thick-walled Helmholtz resonator arrays

For moderately thick-walled resonators, the analysis proceeds as in §6b and 6c but with the replacement $f_{\varepsilon }\mapsto {\overset{ˇ}{f}}_{\varepsilon }$, where the definition for ${\overset{ˇ}{f}}_{\varepsilon }$ is given in Part I (equation (6.9)).

### (e) Descriptions for extremely thick-walled Helmholtz resonator arrays

To obtain an isotropic description for the *extremely thick-walled* resonator configuration discussed in this Part II, the analysis proceeds as in §6b with the replacement $f_{\varepsilon }\mapsto {\bar{\bar{f}}}_{\varepsilon }$ where ${\bar{\bar{f}}}_{\varepsilon }$ is defined in (5.7). As shown in §4a earlier, anisotropic descriptions derived from the regularized system for *extremely thick-walled* resonators are generally intractable. That said, it should be possible to obtain an anisotropic description of the first spectral band alone using (6.7) with the replacement $f_{\varepsilon }\mapsto {\bar{\bar{f}}}_{\varepsilon }$.

## 7. Numerical results

In this section, we compute a broad selection of band diagrams, comparing results from our regularized system (3.3) and asymptotic dispersion equation (4.7) against a full finite-element treatment. We examine the impact of varying the aspect ratio $h=2m/2\ell =(\bar{b}-\bar{a})/2\bar{\ell }$, varying the aperture width $\theta_{\text{ap}}$, and varying the filling fraction f, upon the spectral behaviour of the array, as well as its impact on the Helmholtz resonance frequency. We also evaluate expressions for the effective inverse density and effective inverse bulk modulus (compressibility) for a selection of thin-walled, moderately thick-walled, and *extremely thick-walled* Helmholtz resonator arrays in figures 8 and 9, within the isotropic (6.6) and anisotropic (6.9) approximations given earlier. For reference we consider an air background where B=141.83 KPa and $\rho =1.2041\;\text{kg}\;{\text{m}}^{-3}$.

In figure 3, we examine the band diagram for an array of *extremely thick-walled* resonators near the Γ symmetry point, comparing the result obtained from the system in Part I (with the replacement $h_{\varepsilon }\mapsto {\bar{\bar{h}}}_{\varepsilon }$) against that obtained from the regularized system (3.3) directly. In the former case, figure 3*a* demonstrates unexpected spectral behaviour in the form of band folding effects near k values corresponding to ${\bar{\bar{h}}}_{\varepsilon }\approx 0$. At the cusps of the folded bands, the group velocity $\partial \omega /\partial {\bar{k}}_{Bi}$ is in principle infinite [14], although as shown in figure 3*b*, such features are in fact spurious and are not a feature of the genuine system. Such behaviour demonstrates the need to exercise appropriate caution when calculating band diagrams using multipole methods, although spurious spectral behaviour can be overcome using numerical techniques: by searching for both the zero determinant and vanishing minimum singular value of the matrix system and only considering those values which satisfy both measures.

**Figure 4.**
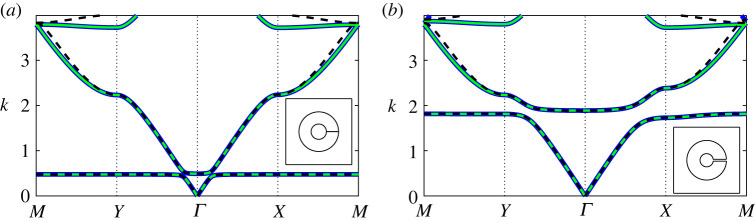
Band diagrams for two-dimensional square arrays of extremely thick-walled Helmholtz resonators comparing the finite-element solution (blue curves) against results obtained using the regularized system (3.3) for dipole L=1 (dashed black curves) and quadrupole L=3 (green curves) truncations. Inset: corresponding fundamental unit cells. Figure (*a*) corresponds to h=100 ($\bar{a}\approx 0.116$) and $\theta_{\text{ap}}=\pi /1024$ and (*b*) corresponds to $\bar{a}=0.1$ and $\theta_{\text{ap}}=\pi /64$. In both figures, we use the truncations $L_{\chi_{n}}=L_{F_{m}}=L_{H_{\varepsilon }}=13$ with $\bar{d}=1$, $\theta_{0}=0$ and $\bar{b}=0.3$. (Online version in colour.)

**Figure 5.**
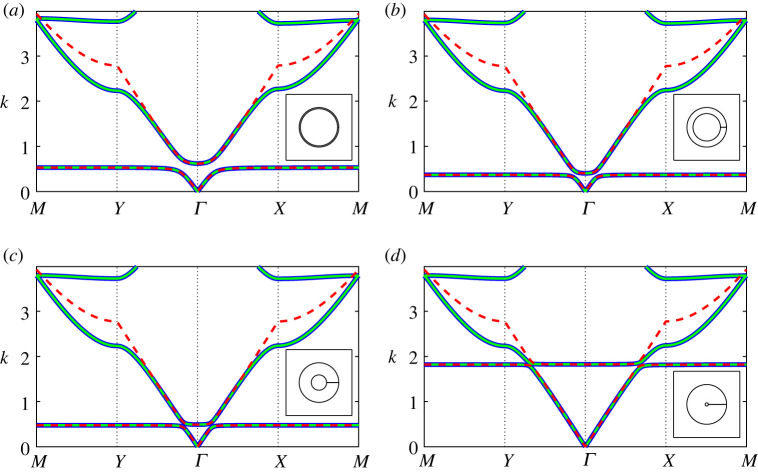
Band diagrams for a two-dimensional square array of extremely thick-walled Helmholtz resonators as the channel aspect ratio h=m/ε is increased: (*a*) h=10, (*b*) h=50, (*c*) h=100 and (*d*) h=150, with fundamental unit cells inset. Multipole results from the regularized system (3.3) are given (green lines) for a system truncation L=3 and truncations $L_{\chi_{n}}=L_{F_{m}}=L_{H_{\varepsilon }}=13$ with the isotropic approximation (4.7) (dashed red lines) superposed, in addition to finite-element results (blue lines). In the above figures, we use $\bar{d}=1$, $\theta_{0}=0$, $\bar{b}=0.3$ and $\theta_{\text{ap}}=\pi /1024$. (Online version in colour.)

**Figure 6.**
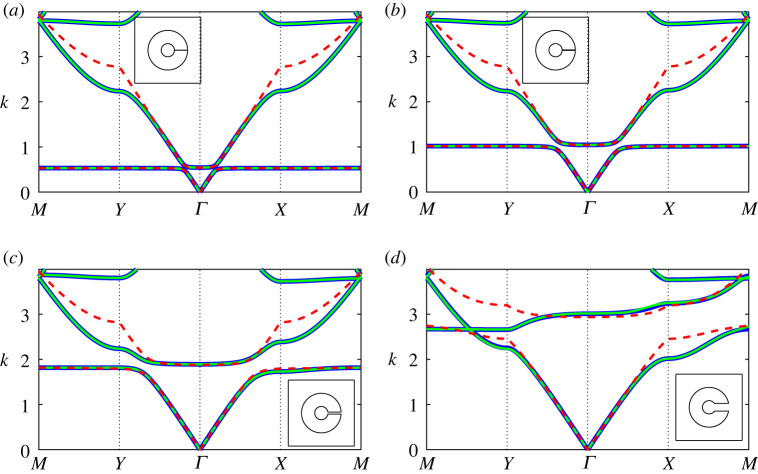
Band diagrams for a two-dimensional square array of *extremely thick-walled* Helmholtz resonators as the aperture half-angle $\theta_{ap}$ is increased: (*a*) $\theta_{ap}=\pi /1024$, (*b*) $\theta_{ap}=\pi /256$, (*c*) $\theta_{ap}=\pi /64$ and (*d*) $\theta_{ap}=\pi /16$, with fundamental unit cells inset. Figure legends and truncation values are identical to those in figure 5; here we use $\bar{d}=1$, $\theta_{0}=0$, $\bar{b}=0.3$ and $\bar{a}=0.1$. (Online version in colour.)

**Figure 7.**
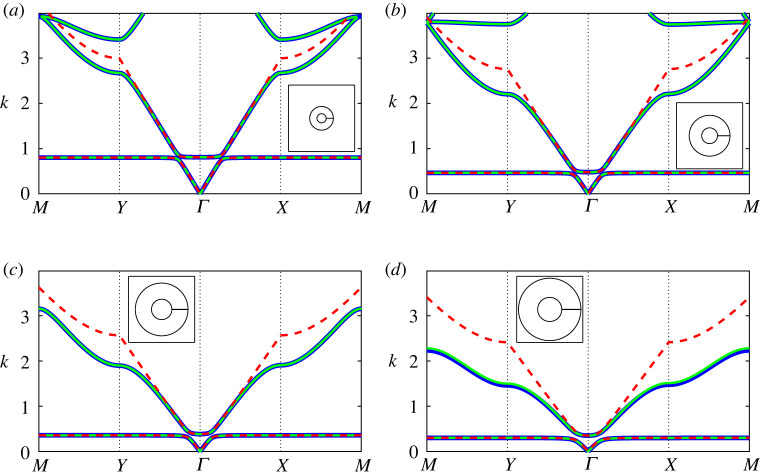
Band diagrams for a two-dimensional square array of *extremely thick-walled* Helmholtz resonators as the filling fraction f is increased: (*a*) f=0.1, (*b*) f=0.3, (*c*) f=0.5 and (*d*) f=0.7, with fundamental unit cells inset. Figure legends and truncation values are identical to those in figure 5; here we use $\bar{d}=1$, $\theta_{0}=0$, h=100 and $\theta_{\text{ap}}=\pi /1024$. (Online version in colour.)

In figure 4, we examine the band diagram for two *extremely thick-walled* resonator configurations, comparing results for the regularized system (3.3) under different truncations against those obtained using finite-element methods. In general, we find that a dipole approximation (dashed black lines) works quite well up to the saddle point frequency of the second band surface, with quadrupole corrections required only for higher frequencies. Hence, in the figures that follow (figures 5–7) we consider a quadrupole system truncation (L=3) for overall accuracy and a dipole system truncations to derive asymptotic descriptions. A significant change is observed in the Helmholtz resonance frequency (equivalently, the maximum frequency of the first band surface) between the two configurations in figure 4, which is attributable to the change in aperture angle. The impact of this parameter is discussed further in figure 10.

**Figure 8.**
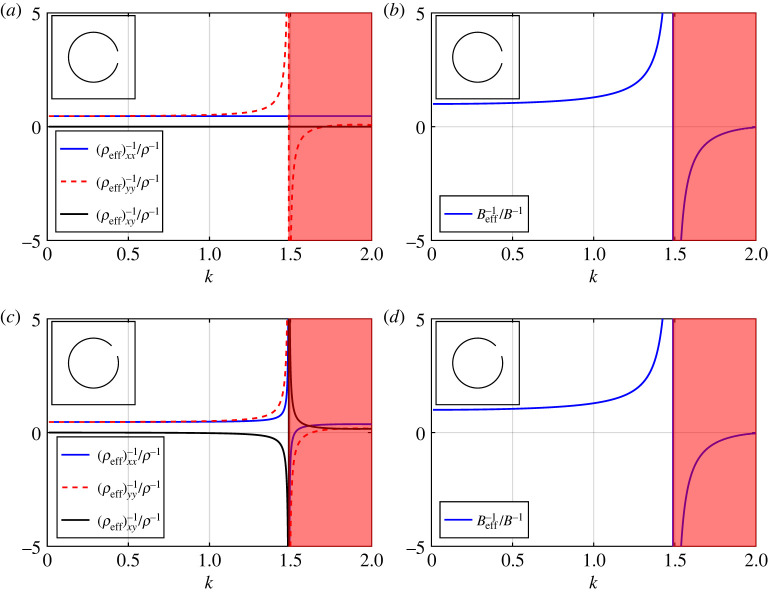
The effective inverse density and effective inverse bulk modulus for a two-dimensional array of thin-walled Helmholtz resonators within an anisotropic approximation (6.9) for (*a*,*b*) $\theta_{0}=0$ and (*c*,*d*) $\theta_{0}=\pi /6$. The shaded red regions denote all k-values above the Helmholtz resonance/cut-off frequency given by vanishing (6.10). Inset: corresponding fundamental unit cells. In the above figures, we use $\bar{d}=1$, $\theta_{\text{ap}}=\pi /12$ and $\bar{b}=0.3$. (Online version in colour.)

**Figure 9.**
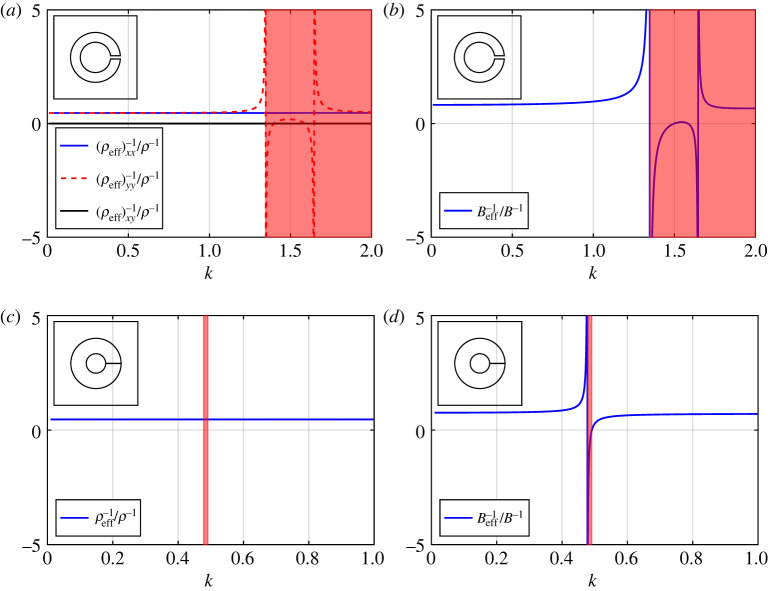
The effective inverse density and effective inverse bulk modulus (*a*,*b*) for a two-dimensional array of moderately thick-walled Helmholtz resonators within an anisotropic approximation (6.9); (*c*,*d*) for an array of *extremely thick-walled* Helmholtz resonators within an isotropic approximation (6.6). Inset: corresponding fundamental unit cells. In (*a*,*b*) the shaded red region denotes all k-values above the Helmholtz resonance/cut-off frequency (6.10), where we use $\bar{d}=1$, $\theta_{0}=0$, $\theta_{\text{ap}}=\pi /48$, h=3, and $\bar{b}=0.3$. In (*c*,*d*) the shaded red region denotes the width of the first band gap as shown in figure 5*c*, where we use $\bar{d}=1$, $\theta_{0}=0$, $\theta_{\text{ap}}=\pi /1024$, h=100 and $\bar{b}=0.3$. (Online version in colour.)

**Figure 10.**
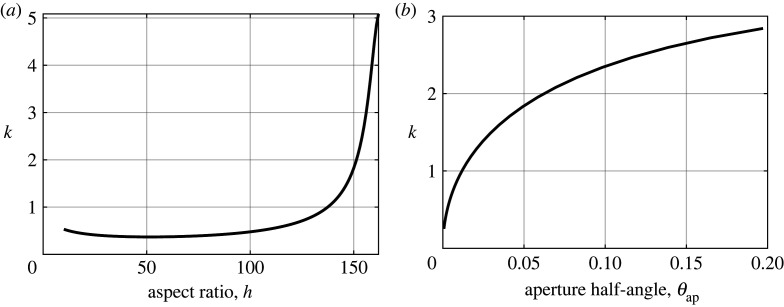
Evaluating the cut-off/Helmholtz resonance frequency (by solving $2{\bar{\bar{f}}}_{\varepsilon }-1\approx 0$) in the *extremely thick-walled* setting as (*a*) the aspect ratio of the resonator neck h=m/ℓ is varied, as shown in figure 5, and as (*b*) the aperture half-angle $\theta_{\text{ap}}$ is varied, as demonstrated in figure 6.

In figure 5, we compute the band diagrams for a *extremely thick-walled* Helmholtz resonator array as the channel aspect ratio h is varied. Here we observe that the multipole system, within a quadrupole approximation, is likewise able to recover the spectral behaviour to excellent accuracy and that the isotropic approximation (4.7) is able to recover the first band, determine the width of the first band gap and describe the second band surface at low frequencies, provided that h is not too large. For all values of h, we find that the isotropic approximation works surprisingly well over the range of the first band surface.

Similarly, figure 6 examines the impact of varying the aperture half-angle $\theta_{\text{ap}}$ on the band diagram for a square array of *extremely thick-walled* resonators. Once more, we find that the multipole system exhibits excellent performance, and that the isotropic approximation (4.7) also performs surprisingly well, provided that the aperture half-angle is not too large. In fact, for $\theta_{\text{ap}}=\pi /16$ we observe that the first and second band surfaces are degenerate along MY (i.e. a band crossing is observed). It is clear that the Helmholtz resonance/cut-off frequency is considerably sensitive to varying aperture angle, which we discuss further in figure 10.

In figure 7, we determine the band diagrams for a *extremely thick-walled* Helmholtz resonator array, as the filling fraction is varied. As in the preceding figures, the multipole treatment works remarkably well, even as the outer resonator wall almost touches the boundaries of the unit cell. This is quite surprising given that an ever-increasing number of multipole orders are required to compute the band diagram for ideal cylinders in the same limit [9]. As before, the isotropic approximation (4.7) is able to recover the first band surface to exceptional accuracy, and is able to extend into the second band surface for moderate filling fractions. Even as the filling fraction approaches the wall-touching limit, the approximation (4.7) is still able to determine the width of the first band gap to suitable accuracy.

In figure 8, we examine the effective inverse density and effective inverse bulk modulus for an array of thin-walled Helmholtz resonators, specifically the anisotropic expression (6.9). In figure 8*a*,*b*, we observe an expected frequency dependence in one component of the effective inverse density matrix as well as for the inverse bulk modulus, when anisotropy is considered, see (6.9). Here, ${\left(\rho_{\text{eff}}\right)}_{yy}^{-1}$ also diverges at the Helmholtz resonance frequency, i.e. at the vanishing of H defined in (6.10). Results for the isotropic approximation are qualitatively similar to those presented here, where $\rho_{\text{eff}}^{-1}/\rho^{-1}$ is given by ${\left(\rho_{\text{eff}}\right)}_{xx}^{-1}/\rho^{-1}$ since frequency dependence is not observed. Similarly, in figure 8*c*,*d*, we observe frequency dependence and divergence at the resonance frequency (6.10) for all entries in ${\left(\rho_{\text{eff}}\right)}_{ij}^{-1}$ as well as for the inverse bulk modulus, when $\theta_{0}=\pi /6$. By rotating the resonator we have reduced the symmetry of the medium, allowing for stronger anisotropy and dispersion. In any case, despite the emergence of anisotropy in the effective inverse density, no changes in sign are observed in the coefficients over the range of the first band surface (i.e. ${\left(\rho_{\text{eff}}\right)}_{xy}^{-1}$ is either zero or negative). This suggests that exotic effects such as negative refraction are not supported on the first band surface for Helmholtz resonator arrays, however we expect that negative refraction is supported at higher frequencies where the correct band curvature is exhibited [15].

In figure 9*a*,*b* we consider the effective inverse density and inverse bulk modulus functions, within an anisotropic description, corresponding to a moderately thick-walled resonator array. These curves are obtained via (6.9) with the replacement $f_{\varepsilon }\mapsto {\overset{ˇ}{f}}_{\varepsilon }$ (results for the isotropic descriptions are not included here as these are qualitatively similar ). As observed in the thin-walled case, we see frequency dependence in both material tensors over the frequency range of the first band surface, which is accompanied by unexpected singular behaviour above the Helmholtz resonance frequency. No firm conclusions may be drawn from this, however, as this behaviour lies outside the region of validity for the expressions. In figure 9*c*,*d*, we consider an *extremely thick-walled* resonator array (i.e. (6.6) with the replacement $f_{\varepsilon }\mapsto {\bar{\bar{f}}}_{\varepsilon }$) which exhibits much more interesting behaviour. In particular, we observe a pole in $B_{\text{eff}}^{-1}$ at low frequencies, where the width of the first band gap may be defined as the interval between the Helmholtz resonance frequency given by $1-2{\bar{\bar{f}}}_{\varepsilon }\approx 0$ and the zero of $B_{\text{eff}}^{-1}$; that is, the first band gap corresponds to the interval where the effective bulk modulus is negative. Once this physical parameter returns to positive values (i.e. upon exiting the band gap) we find that these isotropic descriptions extend well into the range of the second band surface, as seen in figure 5*c*. For reference, we estimate our descriptions (6.6) to hold over the approximate range 0≤k≤1.5 for this example, which supports the assertion that the isotropic description for *extremely thick-walled* resonator arrays appears to be valid over a significantly broader frequency range than for the resonators discussed in Part I.

Finally, in figure 10, to complement the band diagram figures outlined in figures 5 and 6, we solve $2{\bar{\bar{f}}}_{\varepsilon }-1\approx 0$ to track the Helmholtz resonance/cut-off frequency as the aspect ratio h and aperture half-angle $\theta_{\text{ap}}$ are varied. In figure 10*a*, we observe that the cut-off frequency for the first band surface is able to achieve a minimum of k≈0.369 at h≈52 for a fixed $\theta_{\text{ap}}=\pi /1024$, and that as the aspect ratio h becomes large, the cut-off frequency grows larger with polynomial scaling. The possibility of achieving a cutoff frequency minimum, by tuning the wall thickness, may prove useful for those involved in the design of acoustic metamaterials. Likewise, figure 10*b* demonstrates that the resonant frequency decreases with decreasing aperture half-angle, as expected, and scales as $k_{\text{max}}\propto \theta_{\text{ap}}^{1/2}$ as $\theta_{\text{ap}}\to 0$.

## 8. Discussion

In this paper, we have presented a matched asymptotic-multipole expansion procedure for determining the band structure of an acoustic metamaterial comprising a two-dimensional array of Helmholtz resonators that possess large wall thicknesses and narrow neck widths (i.e. high aspect ratios of neck length to neck width). We have also derived a compact dispersion equation which is able to describe the first band surface, first band gap, and frequencies well into the second band surface, over a range of practical settings. In addition, we have outlined a homogenization procedure for thin-walled, moderately thick-walled, and *extremely thick-walled* resonator arrays, presenting analytical forms for the effective inverse density tensor and the effective inverse bulk modulus. The effective response functions derived here extend well beyond the quasi-static limit to higher frequencies, depending on the resonator geometry. Furthermore, we have also derived closed-form representations for the Helmholtz resonance frequency in the *extremely thick-walled* setting.

We demonstrate that the *extremely thick-walled* resonators are able to achieve extremely low Helmholtz resonance frequencies, in contrast to thin- and moderately thick-walled resonators presented in Part I, which has a marked impact on the performance of the array. By incorporating long neck widths 2m into the formulation we provide an additional degree of freedom for controlling the frequency range of the first band surface, indirectly controlling features such as the low-frequency phase and group velocity. Incidentally, the Helmholtz resonance frequency is often approximated in the literature via the form [16, eqn (5.3.12)]

8.1 $$\omega_{\text{max}}\approx \sqrt{\frac{B}{\rho }}\sqrt{\frac{A}{LV}},\;\text{or equivalently},\;k_{\text{max}}\approx \frac{1}{\bar{a}}{\left(\frac{\pi \bar{m}}{\bar{\ell }}\right)}^{-1/2},$$

where A is the total aperture width $2\bar{\ell }$, L denotes the length of the resonator neck $2\bar{m}$, and V denotes the enclosed resonator area $\pi {\bar{a}}^{2}$. The major disadvantage of (8.1) is that the approximation is quite crude, as it treats the neck as distinct from the enclosed volume, and as a result frequently requires correction factors and an 'effective' neck length to recover accuracy. We stress that our Helmholtz resonance expressions in (5.3*b*), (5.6), and in Part I do not require any such corrections.

On comparing results from Parts I and II, we find that anisotropy in both the band structure and in the effective tensors is greatest for thin-walled resonators, with anisotropic effects considerably reduced as we approach the *extremely thick-walled* limit. In fact, we find that *extremely thick-walled* resonators may be treated as *almost-isotropic* media. A core advantage of our matched asymptotic-multipole expansion treatment is that it avoids the need for extensive fully numerical procedures, such a finite-element methods, for determining the low-frequency band structure of acoustic metamaterials. Fully numerical procedures require intensive meshing inside the neck region as it becomes increasingly thin, and although the computational domain is two-dimensional, such requirements can massively increase computation times and resource requirements. By contrast, our asymptotic dispersion equations provide rapidly evaluable closed-form representations for band surfaces over a wide frequency range. It is of interest to extend the methods outlined here to related geometries, such as resonators with long maze-like channels [17] or resonators nested within resonators [18], which is presently under investigation by the authors.

In relation to our homogenization treatment, we emphasize that composite materials and metamaterials have the potential to exhibit generalized constitutive relations [19]. Such behaviour occurs widely across the acoustics and elasticity literature, where these materials are known as *Willis media* [20–22] (or *bi-anisotropic media* in the electromagnetics literature [23]). It would certainly be of interest to investigate, and possibly classify, the existence of Willis coupling effects in resonator arrays in further detail, see [24]. Finally, there are points regarding passivity and causality in resonator arrays, and the behaviour of matched asymptotic expansion solutions, which are worth investigating and this will be reported on by the authors in a forthcoming article.

## Data Availability

This article has no additional data.

## Appendix A. Asymptotic forms of the lattice sums

As discussed in the appendix of Part I, the lattice sums $S_{m}^{Y}$ emerge frequently in the study of periodic media, but are conditionally convergent in their most direct form and so must be regularized in order to retrieve physically meaningful results. The convergent expressions given in Part I are used in all relevant numerical computations, whereas for the asymptotic analysis we follow the procedures outlined in [25,26], to derive forms in the limit as b→0, for example:

A 1 $$\begin{array}{rl} & S_{0}^{Y}∼-\frac{4f}{\pi b^{2}(k_{B}^{2}-1)}+\frac{1}{\pi }\left[-2\gamma_{\text{e}}+\log\left(\frac{f\Gamma {\left(1/4\right)}^{4}}{\pi^{2}b^{2}}\right)\right],\;S_{4}^{Y}∼-\frac{f^{2}\Gamma {\left(\frac{1}{4}\right)}^{8}}{10\pi^{5}b^{4}},\\ & S_{1}^{Y}∼-\frac{4\text{i}fk_{B}}{\pi b^{2}(k_{B}^{2}-1)}{\text{e}}^{\text{i}\theta_{B}}+\left(\frac{\text{i}k_{B}}{\pi }\right){\text{e}}^{\text{i}\theta_{B}},\;S_{6}^{Y}∼\frac{1}{b^{4}}\left[\frac{f^{2}k_{B}^{2}\Gamma {\left(1/4\right)}^{8}}{\pi^{5}}{\text{e}}^{2\text{i}\theta_{B}}+\frac{5f^{2}k_{B}^{2}\Gamma {\left(1/4\right)}^{16}}{960\pi^{9}}{\text{e}}^{-2\text{i}\theta_{B}}\right],\\ \mathrm{and}\; & S_{2}^{Y}∼\frac{4fk_{B}^{2}}{\pi b^{2}(k_{B}^{2}-1)}{\text{e}}^{2\text{i}\theta_{B}}+\left[\frac{k_{B}^{2}\Gamma {\left(\frac{1}{4}\right)}^{8}}{384\pi^{5}}{\text{e}}^{-2\text{i}\theta_{B}}-\frac{k_{B}^{2}}{2\pi }{\text{e}}^{2\text{i}\theta_{B}}\right],\;S_{8}^{Y}∼-\frac{3f^{4}\Gamma {\left(1/4\right)}^{16}}{5\pi^{9}b^{8}}, \end{array}\}$$

where Γ(z) denotes the Gamma function. Although these sums are defined in terms of the lattice period d, we introduce $f=\pi b^{2}/d^{2}$, the cylinder area fraction, here, since the limit of b→0 (with f fixed) is considered in our analysis.
